# The Influence of Transcranial Magnetoacoustic Stimulation Parameters on the Basal Ganglia-Thalamus Neural Network in Parkinson’s Disease

**DOI:** 10.3389/fnins.2021.761720

**Published:** 2021-10-18

**Authors:** Yanqiu Zhang, Mohan Zhang, Zichao Ling, Peiguo Wang, Xiqi Jian

**Affiliations:** ^1^School of Biomedical Engineering and Technology, Tianjin Medical University, Tianjin, China; ^2^Department of Radiotherapy, Cancer Institute and Hospital of Tianjin Medical University, Tianjin, China

**Keywords:** transcranial magnetoacoustic stimulation, Parkinson’s disease, neural network, Hodgkin-Huxley model, numerical simulation

## Abstract

**Objective:** Parkinson’s disease (PD) is a degenerative disease of the nervous system that frequently occurs in the aged. Transcranial magnetoacoustic stimulation (TMAS) is a neuronal adjustment method that combines sound fields and magnetic fields. It has the characteristics of high spatial resolution and noninvasive deep brain focusing.

**Methods:** This paper constructed a simulation model of TMAS based on volunteer’s skull computer tomography, phased controlled transducer and permanent magnet. It simulates a transcranial focused sound pressure field with the Westervelt equation and builds a basal ganglia and thalamus neural network model in the PD state based on the Hodgkin-Huxley model.

**Results:** A biased sinusoidal pulsed ultrasonic TMAS induced current with 0.3 T static magnetic field induction and 0.2 W⋅cm^–2^ sound intensity can effectively modulate PD states with RI ≥ 0.633. The magnitude of magnetic induction strength was changed to 0.2 and 0.4 T. The induced current was the same when the sound intensity was 0.4 and 0.1 W⋅cm^–2^. And the sound pressure level is in the range of −1 dB (the induced current difference is less than or equal to 0.019 μA⋅cm^–2^). TMAS with a duty cycle of approximately 50% can effectively modulates the error firings in the PD neural network with a relay reliability not less than 0.633.

**Conclusion:** TMAS can modulates the state of PD.

## Introduction

Parkinson’s disease (PD) is a common degenerative neurological disease of the nervous system in middle-aged and elderly people. Its incidence is the second highest among neurodegenerative diseases affecting the elderly. Its prevalence among people over 65 is approximately 1.7%. Its incidence and prevalence both increase with age (Zhang et al., 2005). At present, the main therapies for the treatment of early PD are a combination of multiple anti-PD drugs, nerve nucleus damage surgery, and deep brain stimulation therapy. Drug combination therapy needs to gradually increase the drug dose as the course of the disease progresses, and the corresponding side effects such as dyskinesia are more frequent (Angeli and Merkel, 2008). Surgical treatment of nerve nucleus damage is effective, but it is invasive and irreversible. As the disease progresses, some patients require further treatment due to recurrence or even worsening of symptoms (Knovich et al., 2009). In 2002, the United States Food and Drug Administration approved implantable deep brain stimulation (DBS) therapy. The main stimulation targets of this therapy are the internal globus pallidus (GPi) and the subthalamic nucleus (STN) (Hirsch et al., 1988; Aubry et al., 2003). During treatment, it is necessary to drill a hole in the skull, and puncture the brain to implant electrodes to stimulate the GPi or STN. A slight deviation of the electrode stimulation point may induce depression, and there are risks of bleeding, infection, wire breakage. Currently only 1.6–4.5% of severe PD patients are treated with this modality (Morgante et al., 2007; Strutt et al., 2015). Nonimplantable transcranial magnetic stimulation and direct current stimulation have low spatial resolution and are suitable for stimulation of the motor cortex or superficial nerve tissue (Chris et al., 2019).

In 2003, Norton et al. proposed the transcranial magnetoacoustic stimulation (TMAS) method and conducted a theoretical derivation. This technology combines magnetic and focused ultrasound based on the Hall effect, and uses the induced current generated by the coupling of transcranial focused ultrasound and static magnetic field to excite or inhibit neurons in the target area (Norton, 2003). It has the advantages of noninvasiveness, deep brain stimulation, and high spatial resolution. Yuan et al. (2017) studied the influence of TMAS parameters on neuron desynchronization based on Hodgkin-Huxley (H-H) and Hindmarsh-Rose neuron model simulations. The simulation results show that the cycle is similar to the neuron firing cycle and that the duty cycle is 40–70%. Zhang et al. (2018) studied the effect of TMAS current density on the action potential of excitatory or inhibitory cortical neurons based on the Izhikevich model. The results showed that with increasing current density, the firing interval of cortical neurons decreases and the firing frequency increases (Zhang et al., 2018). Wang H. et al. (2019) used TMAS with different spatial peak time average sound intensities under the condition of a static magnetic field intensity of 0.3 T to stimulate the motor cortex of mice. The results showed that when the sound intensity was 25–144 mW⋅cm^–2^, the TMAS induced electric field can be reduced. The EMG signal of the mice is induced under the intensity of the ultrasound (Wang H. et al., 2019). In the same year, Wang Y. et al. (2019) conducted experiments in the hippocampus of TMAS PD mice, and the results showed that TMAS could improve neuroplasticity through postsynaptic regulation. Liu et al. (2020) studied the effect of DBS on the PD state basal ganglia-thalamus (BG-Th) neural network based on the Izhikevich model and used the relay reliability index (RI) to evaluate the stimulation results. The larger the RI, the better the stimulation effect. TMAS can effectively stimulate the activity of brain neurons (Liu et al., 2020). At present, only TMAS numerical simulations and experiments on rats and mice without considering the distribution of induced currents have been carried out. They cannot reflect the state of each neuron in the BG-Th neural circuit, and cannot reflect the effective sound field and its generation under static magnetic field conditions. In mouse or rat animal experiments, because the mouse skull is very thin, it has little effect on the ultrasound transcranial focused sound pressure field, whereas the human skull is approximately 2.33–19.08 mm thick and has heterogeneity, which will distort the ultrasound transcranial focused sound pressure field. Unfavorable phenomena such as phase distortion and defocusing occur, which cause distortion of the focus position and the shape of the TMAS induced current (Ding et al., 2015). In 2006, Yand et al. recorded the frequency of EEG in adult mice at 24–42°C for 60 min, and found that brain tissue damage is reversible when the temperature is less than 40°C (Yang and Qi, 2006).

Transcranial magnetic acoustic stimulation is a method of neuromodulation based on Hall effect coupling TMS and FUS to generate induced currents, which may be accompanied by the stimulating effect of ultrasound during TMAS treatment. Verhagen et al. (2019) stimulated motor association cortex and granular prefrontal cortex of six healthy macaques under pulsed ultrasound conditions with a fundamental frequency of 1.4 MHz, a repetition frequency of 10 Hz, a duration of 20 s, and a time-averaged spatial peak acoustic intensity (I_SPTA_) of 7.2 and 9.5 W cm^–2^, respectively, and showed that changes in neural activity could be induced. Folloni et al. (2019) stimulated the amygdala and anterior cingulate cortex of 11 healthy macaques with pulsed ultrasound with ISPTA of 19.5 and 5.63 W cm^–2^, respectively, at a fundamental frequency of 250 KHz, pulse duration of 30 ms, duty cycle of 30%, and duration of 40 s. The results showed an effect on neural activity in the target area, while there was no effect on non-target (Folloni et al., 2019).

Based on CT images of volunteer’s skull, this paper establishes a TMAS numerical simulation model comprised of human skull, 128-element phase-controlled transducer and permanent magnet. Numerical simulations transcranial sound pressure field, and then coupled with the static magnetic field to obtain the TMAS induction electric field distribution. It stimulates the STN in the BG-Th neural network in the PD state by using the induced currents at the focal point and the acoustic axis, and explored the parameters of the sound pressure field by changing the waveform, duty cycle, and repetition frequency of the transcranial focused ultrasound when fundamental frequency is fixed at 500 kHz. The effect on various neurons in the BG-Th neural network was evaluated, and screening of effective parameters that can make Th respond normally to cortical control motor behavior signals was conducted.

## Models and Methods

The numerical simulations were all performed on a Lenovo Think Station D30 workstation (Lenovo Group Ltd., Beijing, China) with an Nvidia TitanX GPU (NVIDIA Corporation, Santa Clara, CA, United States). Simulations were performed based on computer programming using CUDA C on the platform of Visual Studio Community 2013 (Microsoft Corporation, Redmond, Washington, United States). The simulation of the BG-Th model was carried out in MATLAB (The Mathworks Inc., Natick, MA, United States).

### Sound Pressure Field

#### Numerical Simulation Model

**FIGURE 1:**
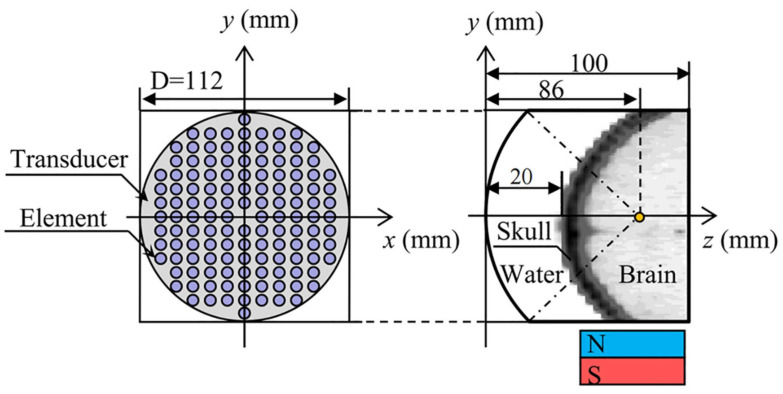
Numerical simulation model of transcranial focusing of a concave spherical phased transducer with 128 elements.

Volunteer’s head CT data (49-year-old male, scanning parameters were 120 kV and 100 mA, scanning thickness was 3 mm) was used to establish a human head, 128-element concave spherical phase-controlled transducer. The numerical simulation model of TMAS transcranial focusing comprised of water and permanent magnets is shown in Figure 1. Among them, the regular distribution array transducer has an opening diameter of 112 mm, a radius of curvature of 86 mm, an element radius of 4 mm, an operating frequency of 0.7 MHz, and a focal depth of 66 mm, which can reach the positions of the STN (Starr, 1999). The numerical simulation area is 112 mm × 112 mm × 100 mm, and the acoustic axis is the z-axis. The spatial step size of the numerical simulation model is dx = dy = dz = 0.25 mm, and the time step size is dt = 10 ns. The boundary of the model is processed by the Mur first-order boundary absorption condition.

#### Sound Wave Equation

The Westervelt acoustic wave nonlinear propagation equation is (Westervelt, 1963; Kang et al., 2008),

(1) $$\nabla^{2}p-\frac{1}{c^{2}}\,\frac{\partial^{2}p}{\partial t^{2}}+\frac{\delta }{c^{4}}\,\frac{\partial^{3}p}{\partial t^{3}}+\frac{\beta }{\rho \,c^{4}}\,\frac{\partial^{2}p^{2}}{\partial t^{2}}=0$$

where ∇^2^ is the Laplacian, *p* (Pa) is the acoustic pressure, *ρ* (kg⋅m^–3^) and c (m⋅s^–1^) are density and sound velocity of the acoustic medium, respectively, and δ = 2*c*^3^α/*ω*^2^ is the acoustic diffusion coefficient where α (dB⋅mm^–1^) is the acoustic attenuation coefficient, β is the nonlinear coefficient, ω = 2πf (rad⋅s^–1^) is the angular frequency, f is the drive frequency of the transducer and t is the irradiation time.

In this paper, the parameters of the skull and brain tissue such as density (ρ), sound speed (c) and attenuation coefficient (α) were obtained from the bone porosity (φ) converted from the Hounsfield unit (H) of the CT images and the calculation method was as follows (Aubry et al., 2003):

(2) φ=1-H/ 1000

(3) $$\rho =\rho_{w\,a\,t\,e\,r}+\left(1-\varphi \right)\times \left(\rho_{b\,o\,n\,e}-c_{b\,o\,n\,e}\right)$$

(4) $$c=c_{w\,a\,t\,e\,r}+\left(1-\varphi \right)\times \left(c_{b\,o\,n\,e}-c_{w\,a\,t\,e\,r}\right)$$

(5) $$\alpha =\alpha_{w\,a\,t\,e\,r}+\varphi^{0.5}\times \left(\alpha_{b\,o\,n\,e}-\alpha_{w\,a\,t\,e\,r}\right)$$

where the *ρ_*bone*_*, *c*_*bone*_ and *α_*bone*_* are the density, speed of sound and the attenuation of cortical skull bone, respectively, and *ρ_*water*_*, *c*_*water*_ and *α_*water*_* are density, speed of sound, and the attenuation of water, respectively. Other constant parameters used in the simulation are shown in Table 1.

**TABLE 1:** Numerical simulation constant parameters.

	ρ (kg⋅m^–3^)	*c* (m⋅s^–1^)	α (dB⋅mm^–1^)	β
Water	998	1,500	0.2	3.50
Cortical skull	1,600	3,200	8	4.40

#### Element Driving Signals

**FIGURE 2:**
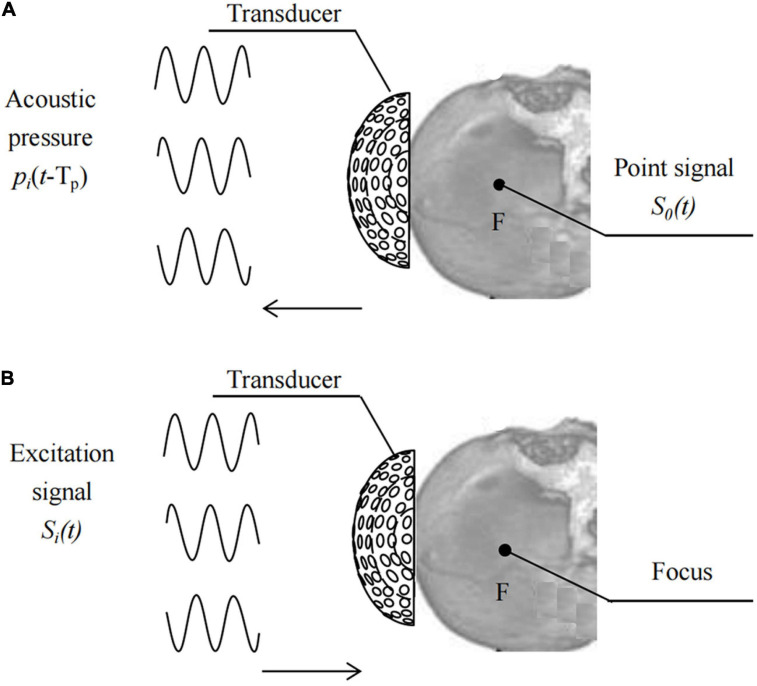
Schematic of **(A)** acquiring signals and **(B)** focusing at F using TR.

Based on the time reversal (TR) method (Ding et al., 2015), place the pulse wave virtual point sound source *S*_0_(*t*) at the target focus F, as shown in Figure 2,

(6) $$S_{0}\,\left(t\right)=\{\begin{array}{c} y\,\left(t\right)\cdot p\,\left(t\right)+b\,n\,T_{s}\leq t\leq n\,T_{s}+T_{1}\\ 0\;n\,T_{s}+T_{1}\leq t\leq \left(n+1\right)\,T_{s} \end{array},0<t<T_{A},n=1,2,3\,\ldots$$

where *p(t)* = *p*_0_sin(2π*ft*) is the instantaneous sound pressure, *p*_0_ is the sound pressure amplitude, *T*_1_ is the sine pulse width, *T*_*s*_ is the repetition period, *T*_1_/*T*_*s*_ is the duty cycle, *T*_*A*_ is the irradiation duration, *b* is the bias parameter, and the pulse function *y*(*t*) is:

(7) y⁢(t)={d1⁢ 2⁢m⁢π≤t<(2⁢m+1)⁢πd2⁢(2⁢m+1)⁢π≤t≤4⁢m⁢π,n⁢Ts<t<n⁢Ts+T1,m=1, 2, 3⁢…

The bias parameter b and the pulse signal *y(t)* jointly determine the pulse ultrasonic wave shape, see Table 2 for details.

**TABLE 2:** Relationship between the parameters and the ultrasonic shape.

Name	*b*	*d*_1_	*d*_2_	
Upper half of sine	0	1	0	
				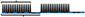
Lower half of sine	0	0	1	
				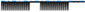
Sine wave	0	1	1	
				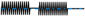
Offset sine wave	1	1	1	
				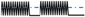

Figures 2A,B show the processes of driving signal acquisition and focusing. Point F was set as the focal targets. Sequentially record the sound pressure signal, *p*_*i*_(*t*-*T*_*p*_), of the sound wave emitted by the point sound source propagating to element i. Reverse each element according to the time series T_*p*_ at different times and obtain the TR signal *p*_*i*_(*T*_*P*_-*t*) of each element in the transducer. T_*p*_ is a time delay sequence containing head information. The relative initial phase delay Δt_*i*_ of p_*i*_ (T_*P*_-t) over a period of time is calculated using the least squares function fitting method, and then the sinusoidal signal amplitude is modulated with the same input sound intensity. The excitation signal of each element after phase adjustment using the numerical fitting method of TR is:

(8) Si⁢(t)={pi⁢(-t+△⁢ti)⁢n⁢Ts≤t≤n⁢Ts+T10 n⁢Ts+T1≤t≤(n+1)⁢Ts⁢ 0<t<TA,n=1,2,3⁢…

### Temperature Field

The temperature distribution was calculated through the Pennes bioheat conduction equation written as (Pennes, 1998; Hallaj and Cleveland, 1999):

(9) $$\rho \,C_{r}\,\partial T/\partial t=r\,\nabla^{2}T+Q-W_{B}\,C_{B}\,\left(T-{\text{T}}_{0}\right)$$

where C_*r*_ [J⋅(kg°C)^–1^] and r [W⋅(m°C)^–1^] are the specific heat and the thermal conductivity of the medium, respectively, T is the transient temperature of the acoustic medium, T_0_ is the initial temperature and set as 37°C in the simulation, Q is the volumetric energy loss which is equal to 2αI, where $I=\frac{1}{t_{p}}\,\int_{0}^{t_{p}}\frac{p^{2}}{2\,\rho \,c}\,dt$ and t_*p*_ is the acoustic wave period, W_*B*_ is the blood perfusion rate and C_*B*_ is the heat capacity of the blood. Other constant parameters used in the simulation are shown in Table 3.

**TABLE 3:** Constant parameters for the temperature field simulation.

	*r* [W⋅(m⋅°C)^–1^]	*C*_*r*_ [J⋅(kg⋅°C)^–1^]	T_0_ (°C)
Water	0.54	4,180	22
Cortical skull	1.30	1,840	37
Brain	0.52	3,700	37

### Electric Field

The Montalibet theoretical equation is (Montalibet et al., 2001):

(10) $$J=\sigma \,v_{z}\,B_{x}\,s\,i\,n\,\left(\omega \,t-\phi \right)/\left(1+t\,a\,n^{2}\,\phi \right)$$

where *J* is the current density; *σ* is the conductivity of the water, scalp and skull, and the *σ* of cerebrospinal fluid and brain tissue are 1.0, 0.33, 0.042, 1.0, and 0.33 S⋅m^–1^, respectively; *v*_*z*_ is the proton vibration velocity; ϕ is the time constant and *B*_*x*_ is the intensity of the static magnetic field perpendicular to the direction of ultrasonic propagation. Because *v*_*z*_=*p*/ρ*c*, the cell level tan*φ* and *φ* are femtoseconds and can be ignored. In summary, the Montalibet theoretical equation can be transformed into:

(11) J≈σ⁢B⁢p/(ρ⁢c)

### Basal Ganglia-Thalamus Neural Network

**FIGURE 3:**
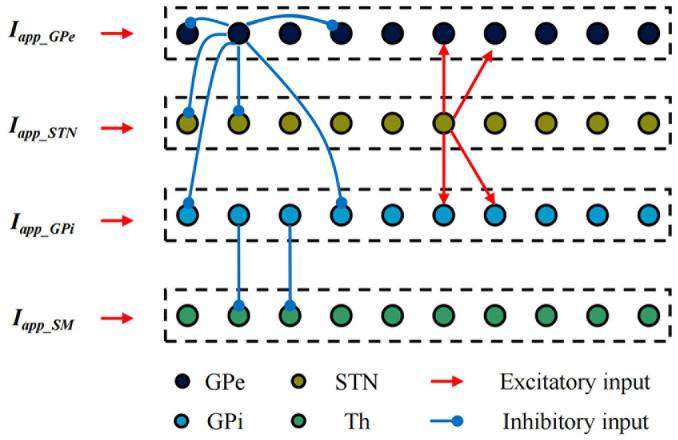
BG-Th neural network model.

Establish single neuron models of the STN, GPe, GPi and Th based on the H-H model. The BG-Th neural network model is constructed as shown in Figure 3 based on the structured sparse connection method, where *I*_*app_GPe*_, *I*_*app_STN*,_ and *I*_*app_GPi*_ are the input currents of other nuclei of the brain to the GPe, STN and GPi, respectively, and *I*_*app_SM*_ is the irregular pulse input current of the sensorimotor cortex to the thalamus. The black, yellow, light blue, and green dots are the Gpe, STN, GPi, and Th neurons, respectively. The red line arrow is the excitatory input current, the blue line point is the inhibitory input current, and the dashed box is a nerve nucleus. The BG-Th neural network model structure adopted from prior study (Rubin and Terman, 2004; So et al., 2012). The synaptic connection of the BG-Th neural network is that each STN neuron excites two GPe and two GPi neurons through excitatory synaptic connections. Each GPe neuron inhibits two GPe, two STN and two GPi neurons through inhibitory synaptic connections. Each GPi neuron inhibits one Th neuron, and each Th neuron receives excitability information from the sensorimotor cortex. Each neuron is assumed to be a spherical cell existing within an isotropic medium. Coupling of ultrasound field and static magnetic field on charged ions in nerve tissues jointly generates current I_TMAS._ This current has been used in studies of neural tissue stimulation (Yuan et al., 2016) and also in simulation studies of H-H neuron models (Lu et al., 2020). The mathematical model of each neuron in the BG-Th neural network model is shown in follow:

(12) $$C_{m}\,\frac{d\,v_{G\,P\,e}}{d\,t}=-I_{L}-I_{N\,a}-I_{K}-I_{T}-I_{C\,a}-I_{A\,H\,P}-I_{G\,P\,e\to S\,T\,N}+I_{a\,p\,p\,\_\,G\,P\,e}$$

(13) $$C_{m}\,\frac{d\,v_{G\,P\,i}}{d\,t}=-I_{L}-I_{N\,a}-I_{K}-I_{T}-I_{C\,a}-I_{A\,H\,P}+I_{S\,T\,N\to G\,P\,i}-I_{G\,P\,e\to G\,P\,i}+I_{a\,p\,p\,\_\,G\,P\,i}$$

(14) $$C_{m}\,\frac{d\,v_{S\,T\,N}}{d\,t}=-I_{L}-I_{N\,a}-I_{K}-I_{T}-I_{C\,a}-I_{A\,H\,P}+I_{S\,T\,N\to G\,P\,e}-I_{G\,P\,e\to G\,P\,e}-I_{a\,p\,p\,\_\,S\,T\,N}+I_{T\,M\,A\,S}$$

(15) $$C_{m}\,\frac{d\,v_{T\,h}}{d\,t}=-I_{L}-I_{N\,a}-I_{K}-I_{T}-I_{G\,P\,i\to T\,h}+I_{S\,M}$$

where *C*_*m*_ = 1 μF⋅μm^–2^ is the membrane capacitance, *v*_*GPe*_, *v*_*GPi*_, *v*_*STN*_ and v*_*Th*_* are the membrane potentials of the GPe, GPi, STN and Th neurons, respectively. *I*_*L*_, *I*_*Na*_, *I*_*K*_, *I*_*T*_, *I*_*Ca*_ and *I*_*AHP*_ are the leak current, sodium current, potassium current, low-threshold T-type calcium current, high threshold calcium current, and after hyper polarization K^+^ current. *I*_*SM*_ is the cortical sensorimotor signal received by the thalamus and can be described by:

(16) $$I_{S\,M}=\{\begin{array}{c} A_{S\,M}\;a/f_{S\,M}<t<a/f_{S\,M}+T_{S\,M}\\ 0\;o\,t\,h\,e\,r\,s \end{array},0<t<T_{A},a=1,2,3\,\ldots$$

where *A_*SM*_* = 3.5 pA μm^–2^ is the amplitude, *T*_*SM*_ = 5 ms is the pulse width, and *f*_*SM*_ is instantaneous frequency. The instantaneous frequencies of the incoming pulses follow a *γ* distribution with an average rate of 14 Hz and a coefficient of variation of 0.2 (So et al., 2012). *I*_*app_i*_ (*i*∈{GPe, STN, GPi}), the positive constant bias currents, which can be viewed as the net synaptic input to these nuclei from other brain regions, *I*_*app_STN*_, *I*_*app_GPe*_ and *I*_*app_GPi*_ are set at 33, 21 and 22 pA μm^–2^ in the healthy state, and 23, 7 and 15 pA μm^–2^ in the PD state, respectively. *I*_*TMAS*_ is the TMAS stimulated current. *I_*m*__?__*n*_* (*m*, *n*∈{GPe, STN, GPi}) represents the synaptic currents from presynaptic cell *m* to postsynaptic cell *n*:

(17) Im→n=gm→n⁢(vm-Em→n)⁢∑jSαj

where *g_*m*__→__*n*_* is the maximum synaptic conductance, *E_*m*__→__*n*_* is the synaptic reversal voltage, $\sum_{j}S_{\alpha }^{j}$ represents the overall synaptic conductance of all presynaptic neurons, *S*_α_ is synaptic variables, and for *I_*GPe*→*GPe*_*, *I_*GPe*→*GPi*_* and *I_*GPe*→*STN*_* are set as:

(18) $$dS_{\text{α}}/dt=2(-S\text{α})H\infty (v-20)-0.04S_{\text{α}}$$

(19) H∞(v)=1/(1+exp((−v−57)/2))

where *H*_∞_ is a step function. For *I_*STN*→*GPe*_*, *I_*STN*→*GPi*_* and *I_*GPi*→*Th*_*, *S*_α_ is set as:

(20) $$dS_{\text{α}}/dt=z_{\text{α}}$$

(21) $$dz_{\text{α}}/dt=0.234u(t)-0.4z_{\text{α}}-0.04S_{\text{α}}$$

where *z*_α_ is the time derivative of the synaptic variable, and if the presynaptic neuron discharge exceeds the threshold of −10 mV at time *t*, the value of u(*t*) is 1; otherwise, it is 0. This paper is based on the random function rand to determine the initial value of the neuron membrane potential and the location of the synaptic connections. The specific parameter settings and variable expressions in the model are shown in Supplementary Appendix. All potentials have units of mV, conductance have the unit of mS⋅cm^–2^, currents have units of μA⋅cm^–2^, concentration have units of mol⋅m^–3^, time constants have units of ms. In addition, *n*′,*h*′,*r*′and [*Ca*]′ are the derivatives of the gating variables, inducing the gating variables to switch between 0 and 1 (So et al., 2012).

### Evaluation of the Stimulation Effects

#### Firing Rates

By counting the number of spikes for each neuron in a nucleus, we can obtain an average rate, *RF*, for every nucleus in the BG-Th neural network,

(22) $$R\,F=f\,i\,r\,e\,s/T_{A}$$

where*fires* is the total number of firings of the neuron during *T*_*A*_.

#### Thalamic Relay Fidelity

The reliability index (*RI*) is another primary index to quantify the Parkinsonian state by measuring the fidelity of Th throughput. *RI* is defined as follows (Rubin and Terman, 2004):

(23) $$R\,I=1-n_{e\,r\,r\,o\,r\,s}/n_{S\,M}$$

where *n*_*SM*_ denote the total number of *I*_*SM*_ pulses, and the *n*_*errors*_ is the inaccurate responses of thalamic neurons to sensorimotor cortex impulses including missed, delay and burst firing corresponding to no excitation, excitation 5 ms after input, and multiple excitations within 25 ms.

In healthy BG-Th, optimum performance of the Th neural network can be achieved, and the *RI* is equal to 1, when each input pulse from the sensorimotor cortex *I*_*SM*_ results in a single action potential in each Th neuron. In addition, in the PD state, 0 < *RI* < 1. Due to the randomness of neuron connections and the value of the initial membrane potential in this paper, all evaluation indicators are averaged five times.

## Results

### Transcranial Magnetic Acoustic Stimulation Induction Electric Field

**FIGURE 4:**
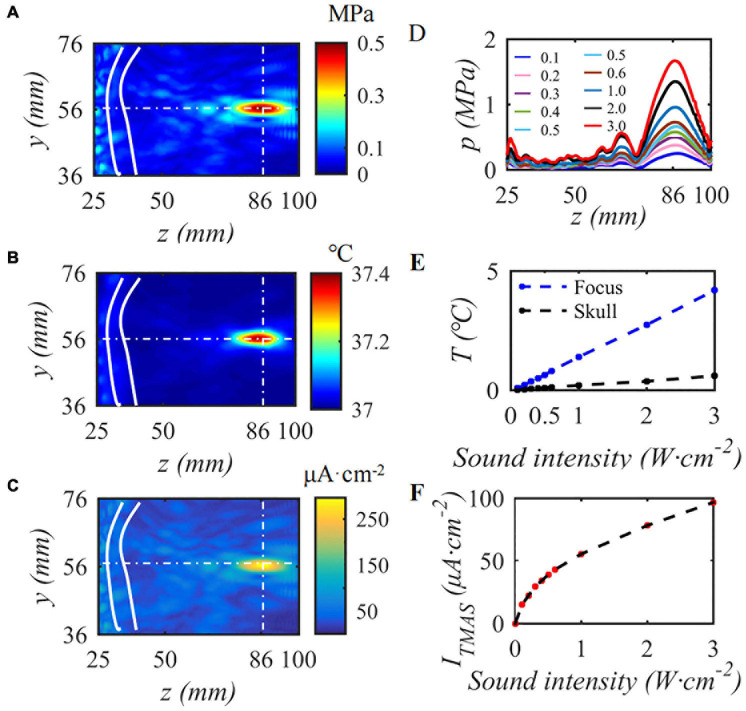
The influence of input sound intensity on TMAS, **(A)** sound pressure field, **(B)** temperature field, **(C)** TMAS-induced field, **(D)** sound pressure curve at the sound axis, **(E)** is the temperature rise at the focus and the skull, **(F)** is the maximum density of the TMAS-induced current at the focal point (*B* = 0.3 T, *t* = 10 s).

This paper first simulates the condition that the input sound intensity of pulsed sinusoidal ultrasonication is 0.3 W⋅cm^–2^, the fundamental frequency is 0.5 MHz, the repetition frequency is 10 Hz, the duty cycle is 50%, and the static magnetic field is 0.3 T. Figure 4 shows the sound pressure field, temperature field and TMAS-induced current density distribution at the geometric focus based on the numerical simulation model of ultrasonic transcranial focusing shown in Figure 1 combined with the TR method. Figures 4A–C show the sound pressure field, temperature field and TMAS-induced current density distribution when the sound intensity is 0.3 W⋅cm^–2^, respectively, and Figures 4D–F are the change curves of the sound axis pressure, temperature rise and induced current density with input sound intensity. The skull is inside the white hyperbola, and the cross dashed line locates the focal point. From Figure 4, after being calibrated by the TR method, it can be accurately focused transcranially. After 10 s of irradiation, the temperature only rose by 0.40°C and there were no hot spots on the skull. The position of the maximum induced current is consistent with the focal position of the sound pressure field. As the ultrasonic input sound intensity increases, the sound pressure at the focal point gradually increases, and the TMAS-induced current density gradually increases. When the input sound intensity is less than or equal to 2.16 W⋅cm^–2^, the temperature rise at the focal point is less than 3°C after 10 s of irradiation, the temperature of the brain tissue is less than 40°C, and there are no hot spots on the skull.

### Stimulate Single Subthalamic Nucleus

**FIGURE 5:**
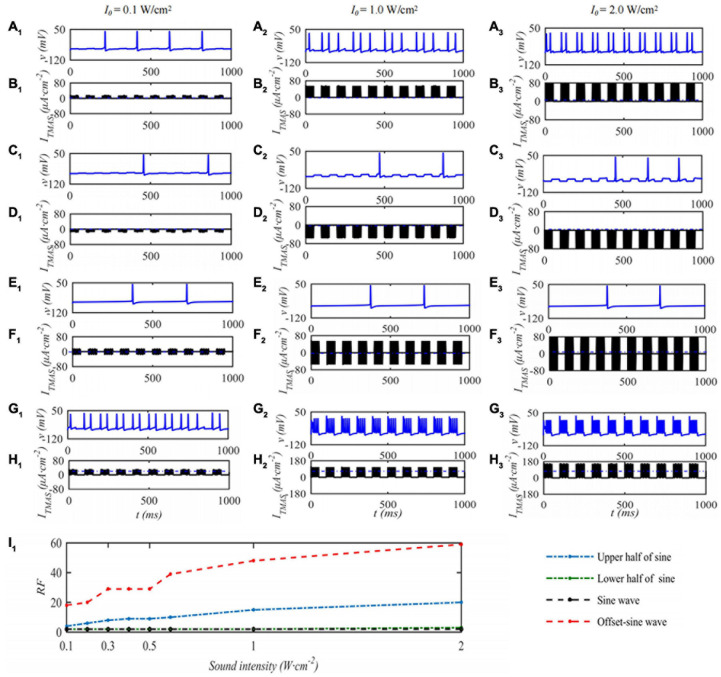
The influence of TMAS input sound intensity on a single STN membrane firing. Columns 1–3 ultrasonic input sound intensity *I*_0_ = 0.1, 1.0 and 2.0 W⋅cm**^–^**^2^, rows 1–4 are the upper, lower half sine wave, sine wave and offset sine wave stimulation. The blue and black curves are STN membrane potential and the TMAS-induced current, **(I_1_)** is the curve of STN neuron firing rate versus TMAS input sound intensity (*f* = 0.5 MHz, *f*_*s*_ = 10 Hz, *T*_1_/*T*_*s*_ = 50%, *B* = 0.3 T).

In this paper, the effect of ultrasound parameters on TMAS treatment was investigated by numerical simulation while keeping the pulsed sinusoidal ultrasound fundamental frequency of 0.5 MHz and the magnetic induction strength of static magnetic field is 0.3 T. Take the TMAS-induced current at the focal point to stimulate a single STN neuron as an example. When the duty cycle is 50%, the repetition frequency is 10 Hz, and the STN neuron membrane potential and firing rate with different TMAS input sound intensities are shown in Figure 5. Columns 1–3 in Figure 5 are the curves of the STN membrane potential and *I*_*TMAS*_ with time under the conditions of an input sound intensity of 0.1, 1.0, and 2.0 W⋅cm^–2^, respectively. Lines 1–2, 3–4, 5–6, and 7–8 in Figure 5 are the pulse upper half sine, pulse lower half sine, pulse sine and bias pulse sine ultrasonic TMAS current stimulator results, respectively. Figure 5I_1_ shows the change curve of the STN discharge rate with ultrasonic input sound intensity. From Figure 5, with the increase of ultrasonic input sound intensity, the STN discharge rate remains basically unchanged after the lower half sine and sine wave stimulation, the STN discharge rate increases after the upper half sine and offset sine wave stimulation, and the STN discharge rate changes dramatically after the bias sine wave stimulation.

**FIGURE 6:**
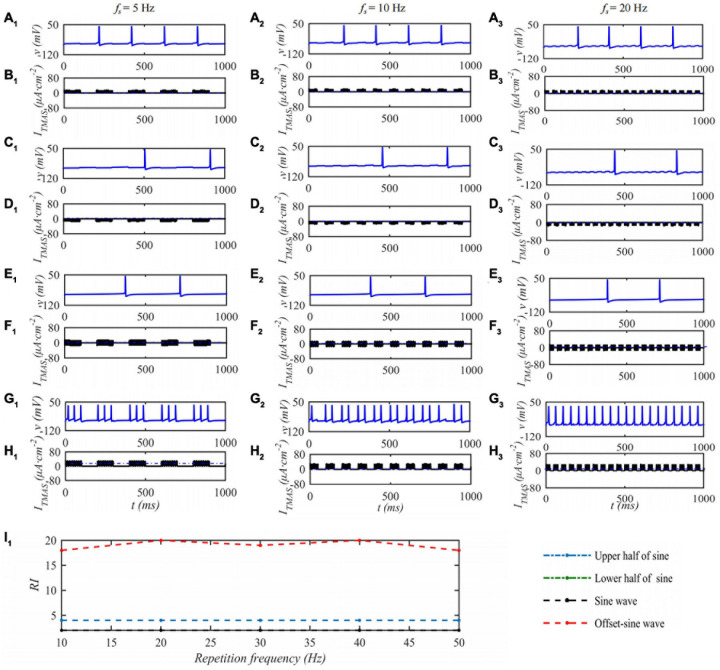
The effect of TMAS repetition frequencies on a single STN discharge. Columns 1–3 ultrasonic pulse repetition frequencies *f*_*s*_ = 5, 10 and 20 Hz, rows 1–4 are the upper, lower half sine wave, sine wave and offset sine wave stimulation, the blue and black curves are STN membrane potential and TMAS-induced current, **(I_1_)** is the curve of STN neuron firing rate versus TMAS repetition frequency (*f* = 0.5 MHz, *I*_0_ = 0.1 W⋅cm^– 2^, *T_1_/T_*s*_* = 50%, *B* = 0.3 T).

Under the condition that the ultrasound duty cycle is 50% and the input sound intensity is 0.1 W⋅cm^–2^, the STN neuron membrane potential changes with the ultrasound pulse repetition frequency of the TMAS stimulation as shown in Figure 6. Columns 1–3 in Figure 6 are the curves of the STN membrane potential and *I*_*TMAS*_ with time under ultrasonic pulse repetition frequencies of 5, 10, and 20 Hz, respectively. Figure 6I_1_ shows the curve of the firing rate with the repetition frequency of the ultrasonic pulse. From Figure 6, with increasing ultrasonic pulse repetition frequency, the STN discharge rate is basically unchanged the pulse upper sine wave, lower half sine wave and sine wave stimulation. After bias sine wave stimulation, the STN discharge rate increases, but as the repetition frequency increases, the STN discharges. The rate of change is small.

**FIGURE 7:**
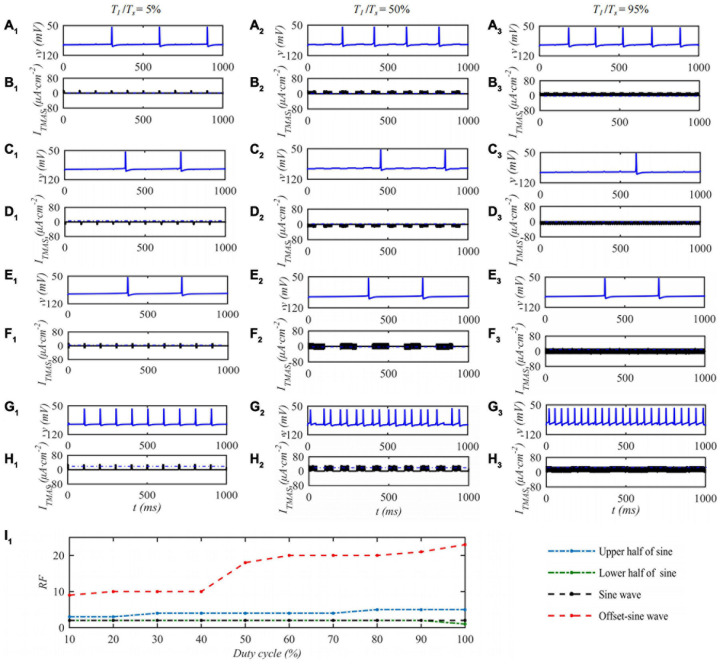
The effect of TMAS duty cycle on the discharge of a single STN. Columns 1–3 Ultrasonic Duty Cycle, *T*_1_/*T*_*s*_ = 5%, 50 and 95%, rows 1–4 are the upper, lower half sine wave, sine wave and offset sine wave stimulation, the blue and black curves are STN membrane potential and the TMAS-induced current, **(I_1_)** is the curve of STN neuron firing rate versus TMAS duty cycle (*f* = 0.5 MHz, *f*_*s*_ = 10 Hz, *I*_0_ = 0.1 W⋅cm^– 2^, *B* = 0.3 T).

When the ultrasonic pulse repetition frequency is 10 Hz and the input sound intensity is 0.1 W⋅cm^–2^, the STN neuron membrane potential changes with the ultrasonic duty cycle of TMAS stimulation as shown in Figure 7. Columns 1–3 of Figure 7 are the curves of the STN membrane potential and *I*_*TMAS*_ with time under duty cycles of 5, 50, and 95%. Figure 7I_1_ shows the discharge rate change curve with the ultrasonic duty cycle. From Figure 7, with the increase of the ultrasound duty cycle, the STN discharge rate is basically unchanged after the lower half sine and sine wave stimulation, the STN discharge rate increases after the upper half sine and bias sine wave stimulation, and the STN discharge rate changes dramatically after the bias sine wave stimulation. Since the upper and lower half sines have opposite effects on neuronal membrane potential excitation and inhibition, sine waves have basically no effect on the neuronal membrane potentials at the considered fundamental frequency of 0.5 MHz. The follow-up study is based on biased sinusoidal pulse ultrasound, which can modulate neurons.

### Stimulate the Subthalamic Nucleus in the Basal Ganglia-Thalamus Neural Network

#### Health and Parkinson’s Disease

**FIGURE 8:**
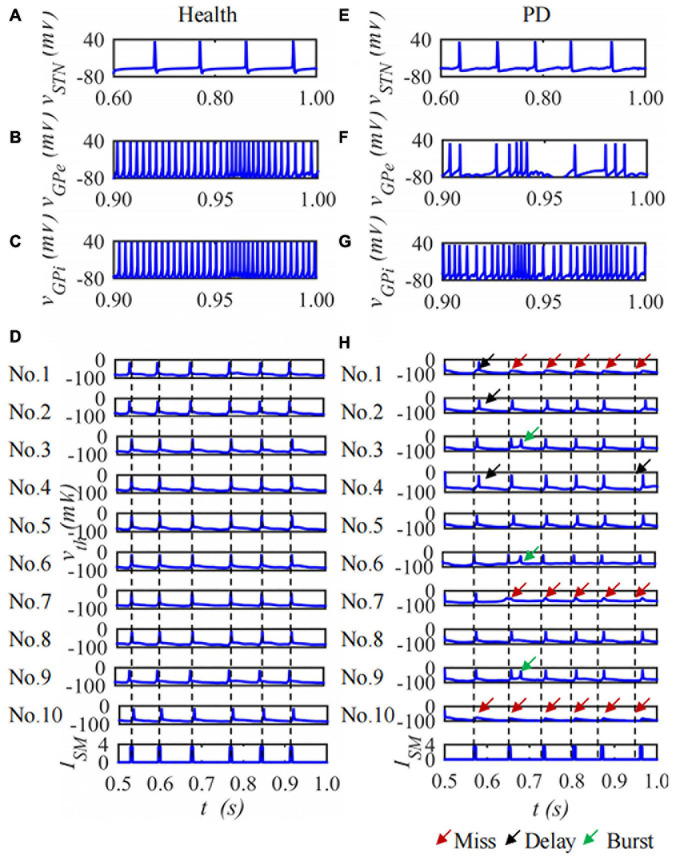
The curve of neuron membrane potential over time, single neuron of STN, GPe, GPi and ten neurons of Th membrane potentials under **(A–D)** health and **(E–H)** PD states.

The BG-Th neural network is constructed based on the H-H model and the structured sparse connection method. Each nucleus contains 10 neurons and the duration is 1,000 ms. The membrane potential of the 1st neuron in the STN, GPe and GPi nuclei and ten neurons of Th membrane potentials in the BG-Th neural network in healthy and PD states changes with time as shown in Figure 8. Figures 8A–C are the membrane potentials of the STN, GPe, and GPi with time in health and Figures 8D–F in PD states. Figures 8D,H are the curves of the membrane potential of 10 neurons in the Th nucleus in the healthy and PD states. The red, black, and green arrows indicate empty firing, delayed firing, and burst firing, respectively. From Figure 8, in the PD state, the firing times of STN and GPi neurons in the BG-Th neural network increase, while the firing times of GPe neurons decrease. In the PD state, Th neurons cannot respond to *I*_*SM*_ one by one.

#### Influence of the Ultrasound Parameters

Under the conditions of a pulse repetition frequency of 10 Hz and an input sound intensity of 0.1 W⋅cm^–2^, the random function rand is used to determine the initial value of the neuron membrane potential and the position of the synaptic connection, and the *RI* value of the Th nucleus after the STN nucleus is stimulated by the TMAS uniform electric field with different ultrasonic duty cycles is shown in Table 4. In Table 4, the first column is the *RI* value corresponding to the original PD state 10 times, and columns 2 to 11 are the *RI* value after TMAS stimulation with different duty cycles. The bold data are larger than the *RI* value in the first column corresponding to the PD state, which means that the stimulus is effective. From Table 4, when the PD *RI* ≥ 0.567, the BG-Th neural network has a corrective effect when using a TMAS stimulating current with a certain duty cycle. When the PD *RI* ≥ 0.667, the TMAS duty cycle is approximately 50%, which can be used for correction in most cases. Follow-up ultrasound parameters were studied for PD status with PD *RI* ≥ 0.667.

**TABLE 4:** *RI* value after TMAS stimulation with different duty cycles (*f* = 0.5 MHz, *f*_*s*_ = 10 Hz, *I*_0_ = 0.1 W⋅cm^–2^, and *B* = 0.3 T).

0% (PD)	10%	20%	30%	40%	50%	60%	70%	80%	90%	100%
0.350	0.333	0.35	0.333	0.300	0.350	0.350	0.333	0.333	0.333	0.333
0.467	0.467	0.467	0.450	0.467	0.450	0.433	0.467	0.467	0.450	0.467
0.500	0.483	0.500	0.467	0.467	0.467	0.416	0.433	0.433	0.417	0.400
0.567	**0.667**	0.517	**0.633**	0.533	0.517	0.533	0.567	0.450	0.567	0.483
0.633	0.617	**0.717**	0.517	0.533	0.567	**0.667**	0.417	0.417	0.400	0.433
0.667	0.617	0.550	0.650	0.567	**0.783**	0.617	0.650	**0.716**	**0.700**	0.550
0.683	**0.767**	**0.717**	0.683	**0.700**	**0.817**	**0.750**	**0.717**	**0.733**	0.683	0.667
0.716	0.450	**0.750**	0.516	0.650	**0.750**	0.483	0.567	0.567	0.167	0.533
0.767	0.750	0.750	0.750	0.633	**0.800**	0.717	0.767	0.733	0.750	0.750
0.883	0.817	0.783	0.750	**0.900**	0.850	0.833	**0.967**	**0.900**	**0.933**	**0.917**

*Data in bold means that the stimulus is valid.*

**FIGURE 9:**
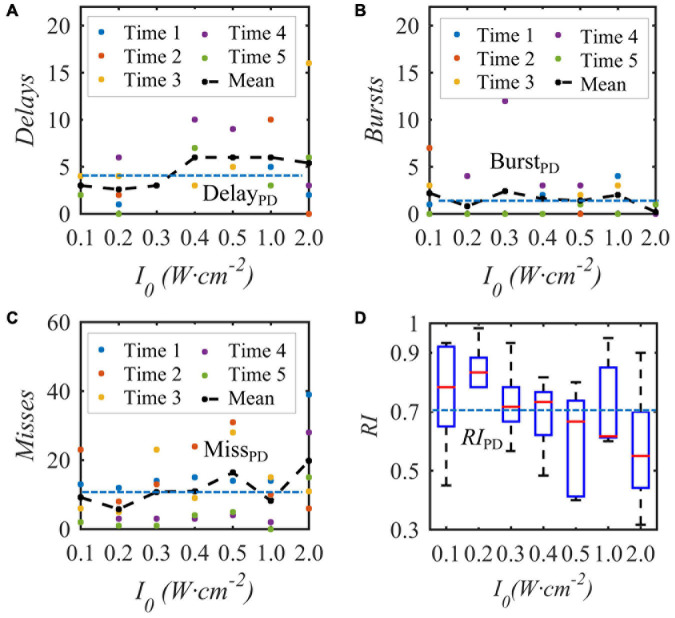
The influence of the input sound intensity of the biased ultrasound TMAS on the abnormal discharge of the Th nerve nucleus in the BG-Th neural network. **(A–C)** Number of delay, burst and missed firing, **(D)** *RI* from Th. The points and curves are the average of single and five times after stimulation, the dashed line is the average of five times in the PD state, (*f* = 0.5 MHz, *f*_*s*_ = 10 Hz, *T_1_/T_*s*_* = 50%, *B* = 0.3 T).

Under the condition that the duty cycle of the bias pulse sinusoidal ultrasound is 50% and the repetition frequency is 10 Hz, uniform TMAS with different ultrasonic input sound intensities at the geometric focus stimulates the STN nucleus in the BG-Th neural network, and the Th discharge result is shown in Figure 9. Figures 9A–C show the delay, burst and missed firing times of Th nerve nucleus after stimulation of TMAS-induced current with an input sound intensity of 0.1–2.0 W⋅cm^–2^, and Figure 9D shows the change curve of *RI* with ultrasonic input sound intensity. The point is a single simulation result, the curve is the fitting curve after five averages, and the point line is the average number of various abnormal responses and Th relay reliability *RI* under the five PD states. Figure 9 shows that as the input sound intensity increases, the abnormal response oscillation increases. When the input sound intensity is between 0.1–0.4 W⋅cm^–2^, the *RI* value after stimulation is greater than the corresponding original PD *RI* value. When the input sound intensity is 0.2 W⋅cm^–2^ with a corresponding maximum induced current density of 22.3 μA⋅cm^–2^, the *RI* value after stimulation is the largest, and TMAS stimulation has the best effect in improving the abnormal response of PD.

**FIGURE 10:**
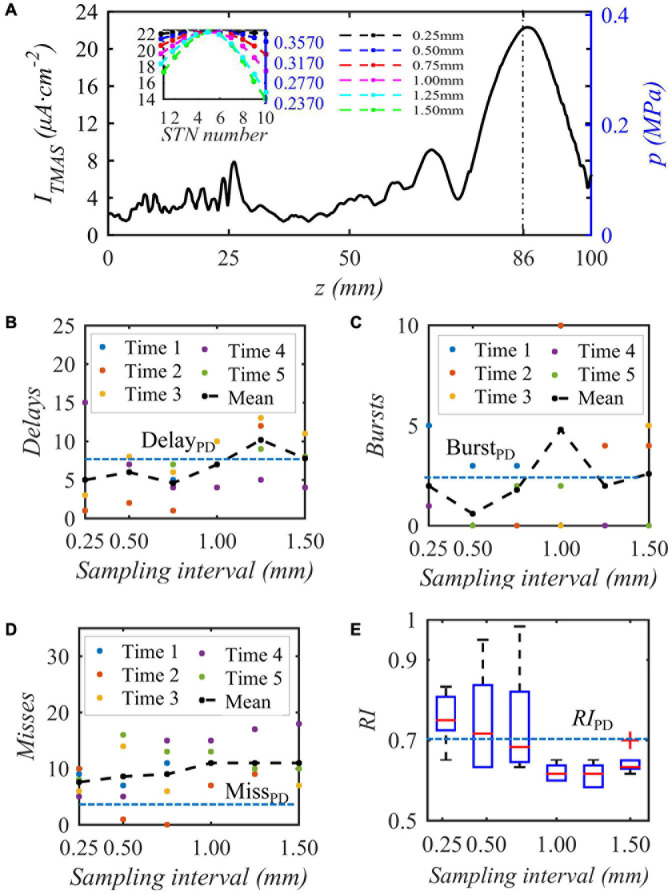
The influence of the biased ultrasound distribution on the abnormal discharge of the Th nerve nucleus in the BG-Th neural network. **(A)** is the acoustic axis sound pressure and the TMAS-induced current density after sampling (block diagram), **(B–D)** Number of delay, burst and missed firing, **(E)** *RI* from Th, (*f* = 0.5 MHz, *f*_*s*_ = 10 Hz, *T_1_/T_*s*_* = 50%, *I*_0_ = 0.2 W cm^– 2^, *B* = 0.3 T).

When the bias pulse sinusoidal ultrasonic input sound intensity is 0.2 W⋅cm^–2^, the duty cycle is 50%, and the repetition frequency is 10 Hz, the acoustic axis sound pressure or induced current density distribution area is as shown in Figure 10A, and then sampling is performed 10 times around the focus point, which is the highest stimulation current. The embedded diagram in the Figure 10A shows the sound pressure and nonuniform TMAS stimulation current received by ten STN neurons, and the delay firing, burst firing and miss firing of Th firing after stimulation are shown in Figures 10B–D. Figure 10E shows the variation curve of *RI* with ultrasonic input sound intensity. From Figure 10, when the sampling interval is 0.25, 0.50, 0.75, 1.00, 1.25, and 1.50 mm, the maximum current difference corresponding to the induced current of TMAS is 0.003, 0.019, 0.044, 0.077, 0.119, and 0.159 μA⋅cm^–2^, respectively. As the TMAS-induced current difference increases, the average number of Th nuclei delayed firing, empty firing, and burst firing gradually increases, and the *RI* gradually decreases. When the maximum current difference of the TMAS-induced current is less than or equal to 0.019 μA⋅cm^–2^, whereas the sampling interval is 0.50 mm, the *RI* value after stimulation is greater than the corresponding original PD *RI* value, and TMAS stimulation can effectively regulate the abnormal response of PD status. When the maximum current difference is less than or equal to 0.019 μA⋅cm^–2^, the corresponding sound pressure level is −1 dB, and the effective nerve stimulation range is less than the −6 dB sound focal range.

#### Influence of the Static Magnetic Field Strength

**FIGURE 11:**
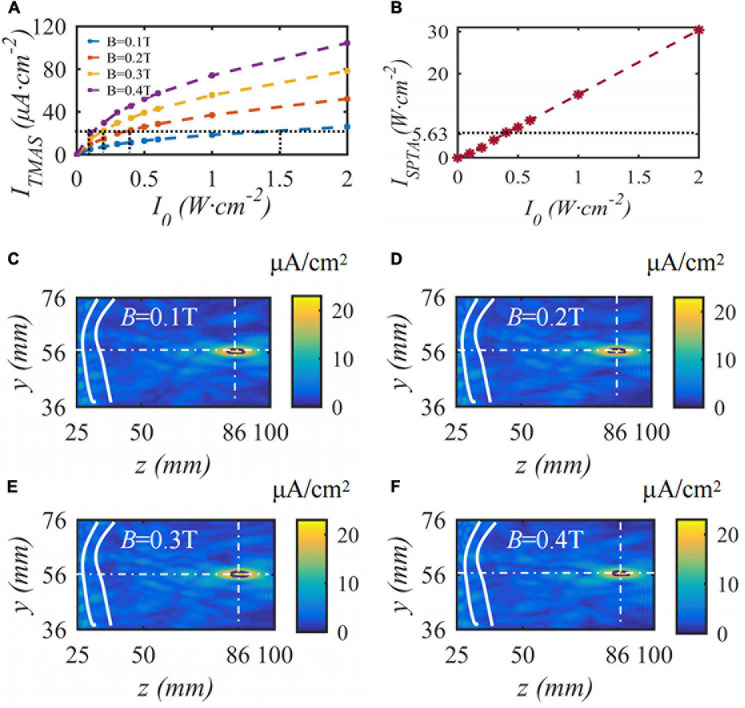
The distribution of induced current density under different static magnetic field strengths, **(A)** is the change curve of induced current density with the input sound intensity, **(B)** is the spatial peak time-averaged sound intensity variation curve with output sound intensity, **(C–F)** are the distribution of induced current density (*J* = 22.3 μA⋅cm^– 2^, *t* = 10 s).

When the fundamental frequency of the bias pulse sinusoidal ultrasound is 0.5 MHz, the duty cycle is 50%, the input sound intensity is 0.2 W⋅cm^–2^, the repetition frequency is 10 Hz, the maximum density of the TMAS-induced current is 22.3 μA⋅cm^–2^, and the current density distributions with different static magnetic field strengths are as shown in Figure 11. Figure 11A shows the change curve of the induced current density with ultrasonic input sound intensity under different magnetic field strength conditions. Figure 11B shows the spatial peak time-averaged sound intensity variation curve with ultrasound output sound intensity at the focus point. Figures 11C–F shows the induced current density distribution under static magnetic field strengths of 0.1, 0.2, 0.3 and 0.4 T. The white curve is the skull, the white cross point is a straight line to locate the focal point, and the black curve is the ultrasonic −1 dB focal range. From Figure 11, to ensure that the maximum induced current density is 22.3 μA⋅cm^–2^, when the static magnetic field intensity is 0.1, 0.2, 0.3, and 0.4 T, the corresponding ultrasonic input sound intensity is 1.5, 0.4, 0.2, and 0.1 W⋅cm^–2^, corresponding to an ultrasonic −1 dB focal length The field size is basically the same. With increasing magnetic field strength, the side lobes of the induced current density gradually increase; however, the TMAS-induced current density distribution is basically the same. When the ultrasound input sound intensity is less than 0.5 W⋅cm^–2^, the focal I_*SPTA*_ is less than 5.63 W⋅cm^–2^, and the stimulation effect of ultrasound in TMAS is negligible.

## Discussion

### Ultrasound Mechanical Effects in Transcranial Magnetic Acoustic Stimulation

The results of this paper show that the TMAS induced current can effectively suppress the PD state under the conditions of magnetic induction strength of 0.3 T and ultrasonic output sound intensity of 0.2 W⋅cm^–2^. The ultrasound focal I_*SPTA*_ under this condition is 2.41 W⋅cm^–2^, which is much smaller than the 5.63 W⋅cm^–2^ with ultrasound stimulation in the literature (Folloni et al., 2019). However, when the magnetic induction strength was less than 0.2 T, the ultrasound output sound intensity was stronger than 0.4 W⋅cm^–2^, the focal I_*SPTA*_ was greater than 4.17 W⋅cm^–2^, and the sound intensity I_*SPTA*_ was close to 5.63 W⋅cm^–2^. Ultrasound can directly cause action potentials in the absence of a magnetic field by several different mechanisms: acoustic radiation force, mechanosensitivity of ion channels, flexoelectricity, cavitation, etc. (Tyler et al., 2008). In this paper, the stimulation effect of induced current is studied according to Montalibet theory, but the direct mechanical effect of ultrasound is not studied. The ultrasound stimulation effect required further analysis.

### The Feasibility of Biased/Rectified Ultrasonic Waveforms

In this study, when performing ultrasonic waveform analysis in TMAS, it was found that at a fundamental frequency of 0.5 MHz, the upper half-sine and lower half-sine of sinusoidal ultrasound TMAS had excitatory and inhibitory effects on neurons, respectively, which would result in a weak stimulation effect on neurons. Since half-sine is difficult to implement in the actual transducer, and bias sine can be generated by superimposing a pulsed square wave signal on the pulsed sine signal, only half-sine is used for the principal analysis of the smaller sine wave effect, and bias sine wave ultrasound TMAS is chosen to explore the effect of stimulation parameters on the BG-TH neural network in the PD state.

### The Limitations of the Used Basal Ganglia-Thalamus Network

The BG mainly includes the striatum, STN, GPe, GPi, substantia nigra pars compacta (SNc) and substantia nigra reticulata (SNr) nuclei. In this study, we simulated the STN, GPe, and GPi nuclei in BG based on the H-H neuron model. The sensory-motor cortex input was considered as pulsed electrical input and the signals from other brain regions were considered as direct current input, and the enhancement and weakening of synaptic connectivity signals due to the absence of dopaminergic neurons in the SNc in the PD state were simulated by changing this direct current input. Each nucleus pulposus is composed of 10 H-H neurons. Although it was shown by Lu et al. (2020) that this neural network can be used for the exploration of the effects of PD state stimulation parameters, in order to consider more comprehensively the effects of TMAS stimulation on the BG-TH neural network, neuronal models of the striatum, SNc, SNr, and sensorimotor cortex could be introduced in the next step. In addition, there is an outlier at the sampling interval of 1.50 mm in Figure 10E, which may be caused by the small volume of the neural network, and the next step is to construct a large-scale BG-TH neural network and increase the number of neurons contained in each neural cluster. In addition, in the spherical cell model, all induced Montalibet currents will cross the neuronal cell membrane. This approximation may lead to a decrease in the threshold value of the calculated TMAS. The next step will be to consider the loss of induced current in the flow through the cell membrane by constructing multi-compartment neuron model.

### The Limitations of Acoustic Modeling

In this study, a non-homogeneous head model is constructed based on real human head CT, and the Westervelt acoustic nonlinear propagation equation be used to simulate the sound pressure field, and the Pennes biological heat conduction equation is sampled to simulate the temperature field. Since vascularity and blood flow are not the focus of attention in this paper, these two factors are not considered in the above model, which may have some effect on the distribution of sound pressure/temperature in the acoustic pressure field/temperature field. In this paper, TMAS modeling simulation was performed based on the cranial CT data of only one volunteer, and the next step will be to investigate the effect of cranial parameters on TMAS PD for the cranial CT data of multiple volunteers.

## Conclusion

Based on the Westervelt acoustic wave nonlinear equation, this paper constructs the ultrasonic transcranial focused sound pressure field, superimposes the uniform static magnetic field, and obtains the TMAS-induced current distribution according to the Montalibet theory. The construction of the PD state BG-Th neural network model is based on the H-H neuron model. The TMAS-induced current is used to stimulate a single STN neuron and the STN nucleus in a neural network, and parameters such as the ultrasonic waveform, sound strength, duty cycle, and repetition frequency are changed according to the firing state and relay reliability judgment stimulus effect. The results show that when the duty cycle of the ultrasonic wave is approximately 50%, the sound pressure field is −1 dB, the static magnetic field strength is 0.3 T and the ultrasonic input sound strength is 0.2 W⋅cm^–2^, The magnitude of magnetic induction strength was changed to 0.2 and 0.4 T. The induced current was the same when the sound intensity was 0.4 and 0.1 W⋅cm^–2^. The TMAS-induced current in the focal range can modulate the PD state with an *RI* not less than 0.633. TMAS could be effective for PD stimulation therapy. These results provide theoretical reference data for the clinically safe and effective treatment of PD by TMAS.

This paper refers to an existing TMAS experiment in rats and mice, and simulates the sound pressure field based on sinusoidal ultrasound before and after modulation and the establishment of the TMAS induction electric field. We propose that when the input sound intensity is between 0.1–0.3 W⋅cm^–2^, TMAS has a modulation effect on PD, which is consistent with the TMAS mouse experiment results of Wang H. et al. (2019). The stimulation effect of other waves needs to be further studied. This paper is only based on the CT data from one volunteer’s skull to conduct TMAS modeling and simulation. The next step will be to explore the influence of skull parameters on TMAS PD based on the CT data of multiple volunteers’ skulls.

## Data Availability Statement

The original contributions presented in the study are included in the article/Supplementary Material, further inquiries can be directed to the corresponding author/s.

## Ethics Statement

This study was reviewed and approved by the Ethics Committee of Tianjin Medical University, China. Written informed consent was obtained from the individual(s) for the publication of any potentially identifiable images or data included in this article.

## Author Contributions

YZ designed the study and wrote the manuscript. MZ and ZL collected the relevant literatures. PW provided the CT data. XJ reviewed and edited the manuscript. All authors read and approved the submitted manuscript.

## Conflict of Interest

The authors declare that the research was conducted in the absence of any commercial or financial relationships that could be construed as a potential conflict of interest.

## Publisher’s Note

All claims expressed in this article are solely those of the authors and do not necessarily represent those of their affiliated organizations, or those of the publisher, the editors and the reviewers. Any product that may be evaluated in this article, or claim that may be made by its manufacturer, is not guaranteed or endorsed by the publisher.
