# Supplementary material for: The Influence of Transcranial Magnetoacoustic Stimulation Parameters on the Basal Ganglia-Thalamus Neural Network in Parkinson’s Disease
Source: Front Neurosci. 2021 Oct 18;15:761720. doi: 10.3389/fnins.2021.761720 (PMC8558679; doi:10.3389/fnins.2021.761720)
Supplement: Supplementary file 1 [file Table_1.DOCX]

|  | STN neuron | GPe / GPi neuron | Th neuron |
| --- | --- | --- | --- |
| *I_L_* | 2.25(*v*+60) | 0.1(*v*+65) | 0.05(*v*+70) |
| *I_K_* | 45*n*^4^(*v*+80) | 30*n*^4^(*v*+60) | 5(0.75(1-*h*)(*v*+75)) |
| *I_Na_* | 37*m_∞_*(*v*)^3^*h*(*v*-55) | 120*m_∞_*(*v*)^3^*h*(*v*-55) | 3*m_∞_*(*v*)^3^*h*(*v*-50) |
| *I_T_* | 0.5*a_∞_*(*v*)^3^*b_∞_*(*r*)^2^*v* | 0.5*a_∞_*(*v*)^3^*rv* | 5*p_∞_*(*v*)^2^*rv* |
| *I_Ca_* | 2*c*^2^(*v*-140) | 0.15*s_∞_*(*v*)^2^(*v*-120) | -- |
| *I_AHP_* | 20(*v*+80)([*Ca*]/([*Ca*]+15)) | 10(*v*+80)([*Ca*]/([*Ca*]+10)) | -- |
| *I_GPe→STN_* | 0.5S*_GPe→STN_*(*v*+85) | -- | -- |
| *I_GPe→GPe_* | -- | 0.5S*_GPe→GPe_*(*v*+85) | -- |
| *I_GPe→GPi_* | -- | 0.5S*_GPe→GPi_*(*v*+85) | -- |
| *I_STN→GPe_* | -- | 0.15*S_STN→GPe_v* | -- |
| *I_STN→GPi_* | -- | 0.15*S_STN→GPi_v* | -- |
| *I_GPi→Th_* | -- | -- | 0.17*S_STN→Th_*(*v*+85) |
| *$n'$* | 0.75(*n_∞_*(*v*)-*n*)/*τ_n_*(*v*) | 0.75(*n_∞_*(*v*)-*n*)/*τ_n_*(*v*) | -- |
| *$h'$* | 0.75(*h_∞_*(*v*)-*h*)/*τ_h_*(*v*) | 0.75(*h_∞_*(*v*)-*h*)/*τ_h_*(*v*) | (*h_∞_*(*v*)-*h*)/*τ_h_*(*v*) |
| *$r'$* | 0.2(*r_∞_*(*v*)-*r*)/*τ_r_*(*v*) | 0.2(*n_∞_*(*v*)-*n*)/30 | (*r_∞_*(*v*)-*r*)/*τ_r_*(*v*) |
| *$c'$* | 0.08(*c_∞_*(*v*)-*c*)/*τ_c_*(*v*) | -- | -- |
| *$[Ca]'$* | 3.75×10^-5^(-*I_CA_*-*I_T_*-22.5×[*Ca*]) | 1×10^-4^(-*I_CA_*-*I_T_*-15×[*Ca*]) | -- |
| *n_∞_*(*v*) | 1/(1+exp((-*v*-32)/8)) | 1/(1+exp((-*v*-50)/14)) | -- |
| *m_∞_*(*v*) | 1/(1+exp((-*v*-30)/15)) | 1/(1+exp((-*v*-37)/10)) | 1/(1+exp((-*v*-37)/7)) |
| *h_∞_*(*v*) | 1/(1+exp((*v*+39)/3.1)) | 1/(1+exp((*v*+58)/12)) | 1/(1+exp((*v*+41)/4)) |
| *a_∞_*(*v*) | 1/(1+exp((-*v*-63)/7.8)) | 1/(1+exp((-*v*-57)/2)) | -- |
| *b_∞_*(*r*) | 1/(1+exp((-*r*+0.4)/0.1))-1/(1+exp(4)) | -- | -- |
| *r_∞_*(*v*) | 1/(1+exp((*v*+67)/2)) | -- | 1/(1+exp((*v*+84)/4)) |
| *c_∞_*(*v*) | 1/(1+exp((-*v*-20)/8)) | -- | -- |
| *s_∞_*(*v*) | -- | 1/(1+exp((-*v*-35)/2)) | -- |
| *p_∞_*(*v*) | -- | -- | 1/(1+exp((-*v*-60)/6.2)) |
| *τ_n_*(*v*) | 1+100/(1+exp((*v*-80)/26)) | 0.05+0.27/(1+exp((*v*-40)/12)) | -- |
| *τ_h_*(*v*) | 1+500/(1+exp((*v*-57)/3)) | 0.05+0.27/(1+exp((*v*-40)/12)) | 1/(1+exp((*v*+41)/4)) |
| *τ_r_*(*v*) | 7.1+17.5/(1+exp((*v*-68)/2.2)) | -- | 0.15(28+exp((-*v*-25)/10.5)) |
| *τ_c_*(*v*) | 1+10/(1+exp((*v*+80/26)) | -- | -- |

**Appendix** BG-Th neural network model parameters and variable expressions.
